# The occurrence of aflatoxins and human health risk estimations in randomly obtained maize from some markets in Ghana

**DOI:** 10.1038/s41598-021-83751-7

**Published:** 2021-02-22

**Authors:** Nii Korley Kortei, Theophilus Annan, Papa Toah Akonor, Seidu A. Richard, Helen Ama Annan, Vincent Kyei-Baffour, Felicia Akuamoa, Princess Golda Akpaloo, Paul Esua-Amoafo

**Affiliations:** 1 Department of Nutrition and Dietetics, School of Allied Health Sciences, University of Health and Allied Sciences, PMB 31, Ho, Ghana; 2 Food Microbiology Division, Council for Scientific and Industrial Research- Food Research Institute, P. O. Box M20, Accra, Ghana; 3 Food Processing and Engineering Division, Council for Scientific and Industrial Research- Food Research Institute, P. O. Box M20, Accra, Ghana; 4 Department of Medicine, Princefield University, P.O. Box MA 128, Ho, Ghana; 5 Food Chemistry and Nutrition Research Division, Council for Scientific and Industrial Research- Food Research Institute, P. O. Box M20, Accra, Ghana; 6 Applied Radiation Biology Centre, Biotechnology and Nuclear Agriculture Research Institute, Ghana Atomic Energy Commission, P. O. Box AE 1, Atomic, Accra, Ghana

**Keywords:** Biotechnology, Microbiology, Environmental sciences, Natural hazards

## Abstract

Maize and its products are most often prone to fungal contamination especially during cultivation and storage by toxigenic fungi. Aflatoxicosis still persist in Ghana despite the numerous education on several ways of its prevention at the farm as well as its adverse health implications which are food safety concerns. A random assessment and human risk analysis was conducted on 90 maize (72 white and 18 colored) samples from markets across all the regions of Ghana. Total aflatoxins (AFtotal) and the constitutive aflatoxins (AFB_1_, AFB_2_, AFG_1_, and AFG_2_) were analyzed by High-Performance Liquid Chromatography (HPLC). Out of a total of ninety (90) samples investigated, 72 (80%) tested positive for AFB_1_ and the contamination levels ranged from 0.78 ± 0.04 to 339.3 ± 8.6 µg kg^−1^. Similarly, AFG2 was detected in only 14 (15.5%) samples, and their values ranged between 1.09 ± 0.03 and 5.51 ± 0.26 µg kg^−1^ while AF total ranged between 0.78 ± 0.04 and 445.01 ± 8.9 µg kg^−1^ constituting approximately 72 (80%). Limits of AFB_1_ and total aflatoxins (AFtotal) for the Ghana Standards Authority (GSA) (5 and 10 µg kg^−1^) and the European Food Safety Authority (EFSA) (2 and 4 µg kg^−1^), were used as checks. A total of 33 (41.25%) samples were above the limits for both. Risk assessments recorded for Estimated Daily Intake (EDI), Hazard Quotient (H.Q), Hazard Index (H.I), Margin of Exposure (MOE), av. Potency, and population risks ranged 0.087–0.38 μg kg^−1^ bw day^−1^, 1.5–6.9, 0.0087–0.38, 3.64–12.09, 0–0.0396 ng Aflatoxins kg^−1^ bw day^−1^ and, 3.5 × 10^–1^–0.015 respectively for total aflatoxins. While ranges for aflatoxins B1 (AFB1) recorded were 0.068–0.3 μg Kg bw^−1^ day^−1^, 2.43–10.64, 0.0068–0.030, 4.73–20.51, 0–0.0396 ng Aflatoxins kg^−1^ bw day^−1^ and, 2.69 × 10^–3^–0.012 for Estimated Daily Intake (EDI), Hazard Quotient (H.Q), Hazard Index (H.I), Margin of Exposure (MOE), Av. potency, and population risks respectively. It was deduced that although there was some observed contamination of maize across the different ecological zones, the consumption of maize (white and colored) posed no adverse health effects on the population of Ghana since computed H.I was less than 1 (< 1).

## Introduction

*Zea mays* (Maize) is a principal cereal and staple for people living in warm climates throughout Asia, Africa, and the Americas who are predisposed to the effects of climate change^1^ in terms of production, consumption and income generation. Forming part of everyday meals, maize, and its products since time immemorial, has been part of the African culture, and so form part of an everyday meal in most homes^2^. It is commonly consumed fresh or processed into cooked or fermented, milled and beverage products^3–5^. It is also extensively used to prepare delectable dishes either singly or in combination with other staples particularly groundnuts or legumes or animal sources of protein to complement each other to combat malnutrition since its protein content is inadequate^23^.

In Ghana, maize is broadly appreciated as a stable crop since it is grown in all agro-ecological zones. More than 50% of rural households cultivate it traditionally under rainfed conditions. Besides, also 16% of urban households are involved in its production. However, there is a yield gap especially in the northern and upper regions. This has ultimately created an imbalance between its production and consumption^6^. Intake levels of approximately 43–46 kg person^−1^ day^−1^ of household consumption of maize in rural subsistence farming communities in Ghana have been reported^7,8^.

Maize is susceptible to fungal infections mainly from *Fusarium* and *Aspergillus* species and consequent contamination with their mycotoxins; fumonisins and aflatoxins^9^ respectively throughout its growth, harvest, transport, and storage^10,11^. A change in climate simultaneously impacts the complex communities of Aflatoxin (AF)-producing fungi by altering the number of AF-producers to change its fungal community’s structure.

Aflatoxins are secondary metabolites, which are naturally occurring contaminants of food and elaborate the toxins under auspicious conditions of temperature, relative humidity, and poor storage conditions. They are now known to be mainly produced by *A*. *flavus*, *A*. *parasiticus*, *Aspergillus nomius* and two different *Emericella* species^12^.

Biochemically, aflatoxins are difurano-coumarin derivatives with a bifuran group joined to the coumarin nucleus and a pentanone ring (in case of AFBs) or a lactone ring (in case of AFGs)^13,14^. AFB1, AFB2, AFG1, and AFG2 are the four most significant AFs among the identified 20. The B-types are produced by *A. flavus* while G-types are produced by *A. parasiticus*^15^. Yu et al.^16^, as well as Yabe and Nakajimam^17^ identified approximately 18 enzymatic steps with at least 25 genes answerable for producing the enzymes and regulating the biosynthesis of aflatoxins process. All the aflatoxins-producing fungi exhibit a great variation in terms of qualitative and quantitative differences in the toxicology abilities that are noticeable attributes by different strains within each fungal species.

Consequently, in sub-Saharan Africa, mycotoxin studies have focused mostly on aflatoxins (in maize and groundnuts) and fumonisins (in maize) while the other potentially dangerous mycotoxins in other foods have received less attention. This is possibly so because maize is a staple food with extensive use that complements groundnuts to combat protein-energy malnutrition typically used in complementary feeding^18^ and so naturally any microorganism and toxins that affect it directly, will be of critical concern. All valuations of exposure implicate maize or groundnuts as the main source of aflatoxins or fumonisins.

The total maize harvest in Africa according to FAO (2017), was estimated at 40 million hectares, with Nigeria being the top producer (16%) followed by Tanzania. Worldwide maize consumption is estimated to be more than 116 million tons with 30% and 21% of the consumption occurring globally and in Sub-Saharan African (SSA), respectively. Approximately 14 countries in SSA consume 85–95% of white maize as their staple food^19^. In most of the developing countries from Africa, there is an increased risk of hepatocellular carcinoma in the presence of hepatitis B virus infection and esophageal cancer being linked to aflatoxins contamination of food^18^.

As emphasized by some previous researchers^20–23^, mycotoxins especially aflatoxins toxicity has always been a topic of contentious interest in the international market and economic development of a country, many of agricultural products are often rejected due to excessive contaminations (beyond specific thresholds of host countries). This is evidenced in previously published works on aflatoxins and cereals in Ghana which revealed some tenacity and unsatisfactory trend of contamination^24–28^.

To overcome this problem, many countries have set standard safety levels of aflatoxins in food and food products and animal feed to ensure quality. Furthermore, several attempts have been made to educate the populace and stakeholders on the preventive practices and impacts of these mycotoxins on health^29^ chiefly attributable to non-compliance to Good Management Practices (GMP), Good Agricultural Practices (GAP) and Good Hygienic Practice (GHP).

It was hypothesized that maize grains meant for consumption and sold on markets across Ghana did not contain aflatoxins. The objective of this study was, therefore, to assess the potential exposure to aflatoxin through consumption of commercial maize products (market maize), we conducted a cross-sectional assessment of market maize contamination.

## Materials and methods

### Study area

**Figure 1:**
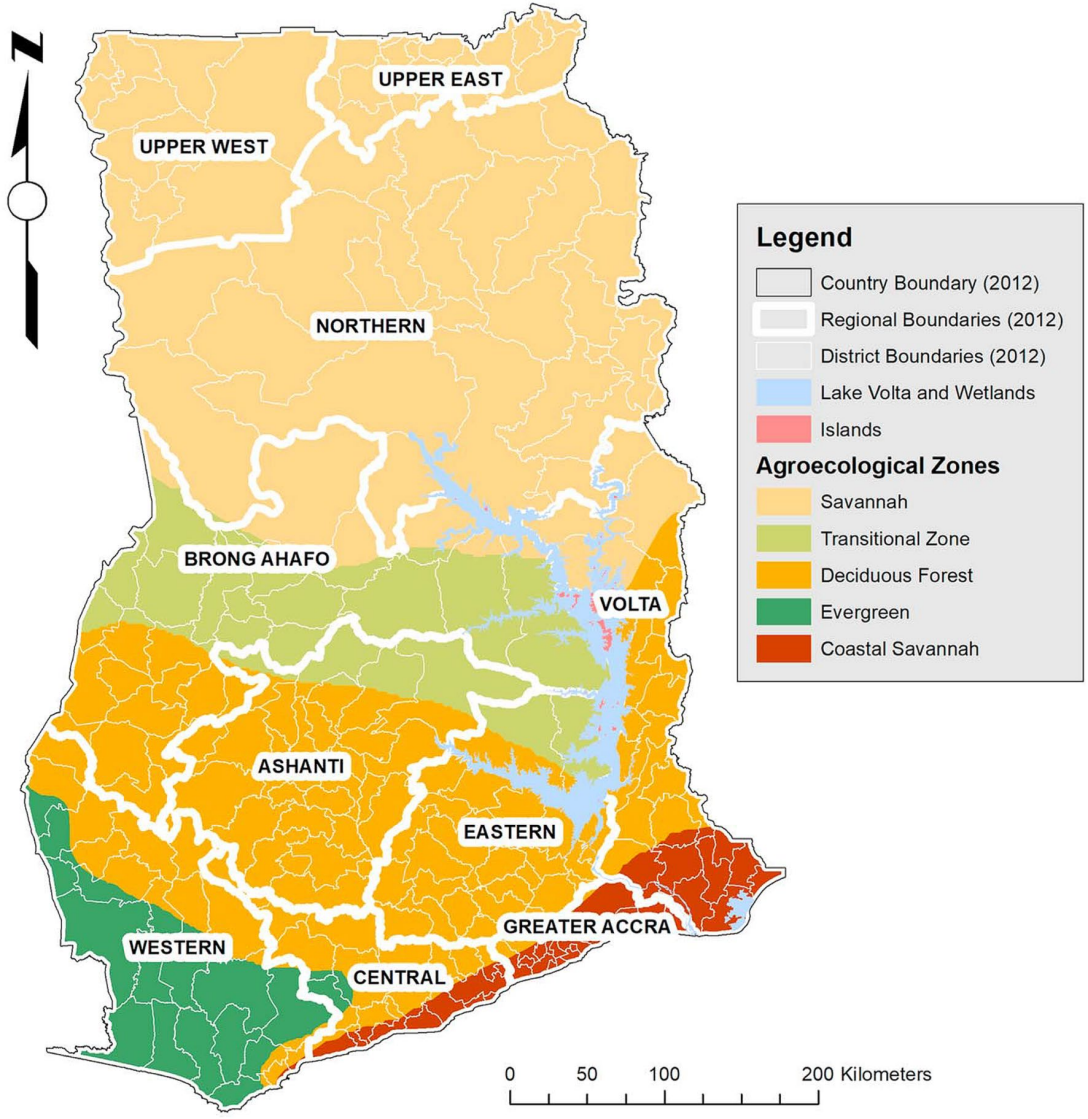
Map of Ghana showing regions of sampling (Adapted from Abbam et al.^33^).

On the Gulf of Guinea in West Africa, is located in Ghana. It covers about 23,884,245 ha of land and water area between latitudes 4°N and 11°N and longitudes 4°W and 2°E^30,31^. The country is demarcated into 10 regions and 216 districts, categorized into five main agro-ecological (Coastal Savannah, Evergreen, Deciduous Forest, Transitional, and Savannah) zones (Fig. 1). An estimated 24,658,832 people were counted across the whole country during the 2010 Ghana Population and Housing Census^32^.

The three northern regions (Upper West, Upper East, and Northern) are predominantly agro-ecologically Savannah. Ashanti, Brong-Ahafo, and Eastern regions are mainly transitional and deciduous forest areas. The western region is mostly Evergreen and Deciduous Forest and highly economically active. Greater Accra (predominantly Coastal) and Ashanti (predominantly Deciduous Forest) are the most developed and urbanized regions and the lowest in terms of agricultural activity. The Volta region cuts across three agro-ecological (coastal, deciduous forest, and savannah) zones. The central region, primarily deciduous forest and coastal, is the fourth poorest region. Fishing and agriculture are the main economic activities. The five principal maize growing areas are in the Northern, Brong‐Ahafo, Ashanti, Central and Eastern Regions.

### Sample collection

To collect a representative data set, we first obtained the list of villages in each district from the Regional Directorate of the Ministry of Agriculture. From each district, an average of 9 villages (Table 1) were then randomly selected. The maize sellers in each market were conveniently sampled where about one kilogram (1 kg) of raw maize samples were purchased concurrently within the period of February to September 2018 and grouped into 2 categories (white and colored maize) (Tables 3, 4, 5, 6 and 7) Twenty (20) grams each of maize samples were fetched and kept in sterile bags in ice chests and sent to the laboratory within the same day in a vehicle where they were stored in a deep freezer at − 20 °C until ready for chemical analysis^35^.

**Table 1:** Geographical locations and some attributes of the origin of samples.

Region	No. of samples	Agro-ecological zones	Rainfall (mm)	Temperature (°C)	Coordinates
Greater Accra	9/90	Coastal Savannah	800–1000	26.6	5.8143° N, 0.0747° E
Central	9/90	Deciduous Forest	1400–1600	26.7	5.5608° N, 1.0586° W
Western	8/90	Evergreen	1800–2000	25.9	5.3902° N, 2.1450° W
Eastern	12/90	Deciduous Forest	1400–1900	25.9	6.2374° N, 0.4502° W
Ashanti	11/90	Deciduous Forest	1200–1400	26.3	6.7470° N, 1.5209° W
Brong-Ahafo	9/90	Transitional zone	1400–1600	23.9	7.9559° N, 1.6761° W
Volta	7/90	Coastal Savannah/Deciduous forest	1000–1400	26.2	6.5781° N, 0.4502° E
Northern	10/90	Savannah	1000–1200	27.9	9.5439° N, 0.9057° W
Upper East	7/90	Savannah	800–1000	28.3	10.7082° N, 0.9821° W
Upper West	8/90	Savannah	1000–1200	27.8	10.2530° N, 2.1450° W

### Extraction of samples

AFB_1_, AFB_2_, AFG, and AFG_2_ were extracted from samples according to the European Committee for Standardization (CEN) official method EN14123^34^ for aflatoxin extraction. Methanol in water (200 ml) (8 + 2) and 5 g NaCl were used to extract 25 g of sample. Hexane (100 ml) was added to samples containing more than 50% fat. Mixture was homogenized for 3 min at 3000 rpm (2 min) and 3500 rpm (1 min). The extracts were filtered and 10 ml of filtrate added to 60 ml of phosphate buffer saline (PBS) for solid-phase extraction using a pre-conditioned immune-affinity columns specific for AFB_1_, AFB_2_, AFG_1_, and AFG_2_. The 70 ml filtrate-PBS mixture was loaded onto the pre-conditioned column and allowed to elute by gravity at a flow rate of 1 ml min^−1^. This was followed by a cleanup with 15 ml distilled water at a flow rate of 5 ml min^−1^. Aflatoxins were eluted in two steps into a 5 ml volumetric flask with 0.5 ml followed by 0.75 ml of methanol (HPLC grade) and allowed to elute by gravity. Deionized water was used to make up volume of eluates to 5 ml and eluate vortexed and 2 ml pipetted into HPLC vials for quantification^35^.

### HPLC parameters

Injection volume: 10 μl flow rate: 1 ml min^−1^, column temperature: 35 °C, excitation wavelength: 360 nm, emission wavelength: 440 nm, mobile phase composition: water/acetonitrile/MeOH (65:15:20 v/v/v), post-column derivatization: Kobra cells. HPLC Column Specification Spherisorb ODS1- Excel (4.6 mm × 25 cm), 5 μm particle size, 250 A pore size.

LOD = Limit of detection.

LOQ = Limit of quantification.

ACN = Acetonitrile.

MeOH = Methanol.

LOD calculation = 3 * standard deviation/slope.

LOQ calculation = 3 × LOD.

Supplier of Column R- Biopharm, Block 10 campus, West Scotland.

Science Park, Acre Road, Glasgow, Scotland G20 OXA^35^.

#### Analysis of samples

The aflatoxins (by *Aspergillus flavus* and *A. parasiticus*) levels in the samples were determined by High-Performance Liquid Chromatography HPLC (Agilent 1260 Series, OpenLab software, X-bridge column) (250 mm × 4.6 mm, i.d., 5 μm), USA with fluorescence detector and post-column derivatization using Kobra cells to generate bromine electrochemically at the CSIR- Food Research Institute, Ghana. LOD for AFB1, AFB2, AFG1 and AFG2 were 0.20 μg kg^−1^, 0.17 μg kg^−1^, 0.26 μg kg^−1^ and 0.36 μg kg^−1^ respectively (Table 2)^35^.

**Table 2:** Limits of Detection and Quantification (LOD & LOQ) of aflatoxins AFB_1_, AFB_2_, AFG_1_, AFG_2_ and Total aflatoxins (µg/kg) measured by HPLC.

Aflatoxin	Limits	Amount (µg/kg)
AFB1	LOD	0.20
	LOQ	0.60
AFB2	LOD	0.17
	LOQ	0.51
AFG1	LOD	0.26
	LOQ	0.78
AFG2	LOD	0.36
	LOQ	1.08

*LOD* limit of detection, *LOQ* limit of quantification.

### Limit of detection/quantification (LOD/LOQ)

Limit of detection and quantification (LOD/LOQ) of the HPLC were estimated by making a calibration curve around the least standard used for spiking, 5 µ kg^−1^ (lowest concentration range of calibration curve). Blank did not produce any signal, so the LOD and LOQ were calculated as;

1 $$ {\text{LOD}} = {3}*{\text{standard}}\;{\text{deviation}}/{\text{slope}}. $$

2 $$ {\text{LOQ}} = {3}*{\text{LOD}}. $$

### Measurement accuracy

Spiking of pure aflatoxin standard solution was done according to method described by Kortei et al.^35^ to ensure measurement accuracy of analysis. Three levels spiking were done at the lower, mid and upper concentration range of the calibration curve concentrations (5 ppb, 15 ppb and 30 ppb). Spike volumes of pure standards were calculated as;

3 $$ \left[ {{\text{Sample}}\;{\text{weight}}\;\left( {\text{g}} \right)*{\text{spike}}\;{\text{concentration}}\;\left( {{\text{ppb}}} \right)} \right]/\left[ {{\text{Concentration}}\;{\text{of}}\;{\text{standard}}\;\left( {{\text{ug}}/{\text{ml}}} \right)} \right]. $$

Spike volumes were distributed evenly on aflatoxins free sample (blank) and spiked sample analysed for percentage recovery which was calculated as;

4 $$ [({\text{Concentration}}\;{\text{measured}}\;{\text{in}}\;{\text{spike}} - {\text{concentration}}\;{\text{measured}}\;{\text{in}}\;{\text{blank}})/({\text{spiked}}\;{\text{amount}})]*{1}00 $$

### Measurement precision

Repeatability and intermediate precision analyses of an internal reference material (IRM) was used to ensure measurement precision of the method. For repeatability analysis, 10 parallel extractions of the IRM was done by the same analyst at the same time using the same HPLC and the relative standard deviation among results calculated. For intermediate precision, 10 extractions of the IRM were done at different days by different analysts and the relative standard deviation among results calculated^35^. The relative standard deviations were calculated as; [Standard deviation/mean] * 100.

### Required performance criteria for accuracy and precision

#### Repeatability

Relative standard deviation among repeatable results should be less than 15%.

#### Intermediate precision

Relative standard deviation among results obtained under intermediate precision conditions should be less than 20%.

#### Recovery

Percent recovery of measurement procedure should be in a range of 80–120%.

#### Limit of detection

The limit of detection should be less than 1 ug kg^−1^ for all aflatoxins.

#### Limit of quantification

The limit of Quantification should be less than 3 ug kg^−1^ for all aflatoxins.

#### Linearity

Linearity from regression curve should be 0.99 (B1, B2, G1) and 0.98 (G2)^35^.

## Experimental data

### Repeatability

Relative standard deviation was; B1 = 5.5%; B2 = 6.7%; G1 = 7.4%; G2 = 12.1% and Total aflatoxins = 5.2%.

Intermediate Precision (Reproducibility): Relative standard deviation was; B1 = 13.2%; B2 = 13.4%; G1 = 13.7%; G2 = 12.2% and Total aflatoxins = 11.9%.

Recovery: Percent recovery of measurement procedure was; Low concentration: B1 = 107%; B2 = 87.2%; G1 = 113.4%; G2 = 112.8% and Total aflatoxins = 108.2%.

High concentration: B1 = 102.6%; B2 = 101.6%; G1 = 104.2%; G2 = 104.4% and Total aflatoxins = 103.3%.

Linearity: Linearity from regression curve was; B1 = 0.991; B2 = 0.997, G1 = 0.994; G2 = 0.995.

Human health risk assessment of exposure to total aflatoxins via consumption of cereals.

### Exposure estimation

Calculation of the Estimated Daily Intake (EDI) was done by using the mean levels of aflatoxins obtained in maize samples, the daily intakes of the same samples^6^, and the average body weight. The EDI for mean aflatoxins was calculated according to the following formula and expressed in μg kg^−1^ of body weight/day (μg kg bw^−1^day^−1^)^36^.

5 $$ {\text{EDI}} = \frac{{{\text{daily}}\;{\text{intake}}\left( {{\text{food}}} \right) \times {\text{mean}}\;{\text{level}}\;{\text{of}}\;{\text{Aflatoxin}}}}{{{\text{average}}\;{\text{bodyweight}}}} $$

Daily intake of maize in Ghana according to MoFA^6^ is 42.5 kg day^−1^.

### Estimation of hazard quotient (HQ)

Hazard Quotient (HQ) is otherwise referred to as the Non-Carcinogenic Effect of the toxin. The non-carcinogenic effect of the individual toxin is designated by hazard quotient (HQ) as described by Kortei et al.^25^. The HQ was estimated using Eq. (6):

6 $$ {\text{HQ}} = \frac{{{\text{EDI}}}}{{{\text{RFD}}}} $$

where EDI and rfD are average daily dose and reference dose respectively.

$$ {\text{rfD}} = {\text{ TD}}_{{{5}0}} $$

### Estimation of Hazard Index (HI)

The Hazard Index (HI) was calculated according to the below-mentioned formula, by dividing the EDI by TD_50_, divided by a safety factor of 50,000. TD_50_ is the dose (ng kg^−1^ body weight^−1^ day^−1^) required to induce tumors in half of the test animals that would have remained tumor-free at zero doses as described by Ismail et al.^37^ and Ishikawa et al.^38^.

7 $$ HI = \sum\limits_{{{\text{n}} = 0}}^{1} {\frac{{\left( {{\text{EDI}}/{\text{TD}}_{{{5}0}} } \right)}}{50000}} $$

### Population risk characterization for aflatoxins

Risk characterization for genotoxic and carcinogenic compounds such as aflatoxins is based on the margin of exposures (MOEs), which was calculated by dividing the Benchmark dose lower limit (BMDL) for aflatoxins- 400 ngkg^−1^ bw day^−1^ by toxin exposure^39^

8 $$ {\text{MOE}} = \frac{{{\text{Benchmark}}\;{\text{dose}}\;{\text{lower}}\;{\text{limit}}}}{{{\text{EDI}}\;\left( {{\text{Exposure}}} \right)}} $$

In cases where MOEs were lower than 10,000, a public health concern is indicated which implied that aflatoxin exposures above 0.04 ngkg^−1^ bw day^−1^ (as obtained by dividing 400 ngkg^−1^ bw day^−1^ by 10,000) represented a risk of public health concern^40^.

### Estimated liver cancer risk due to consumption of maize

The estimated liver cancer risk for Ghanaian adult consumers was calculated for aflatoxins because the ingestion of the toxin can be traced to the development of liver cancer^11,39^. This involved estimating the population cancer risk per 100,000 which was obtained by multiplying the EDI value with the average hepatocellular carcinoma (HCC) potency figure from individual potencies of HBsAg-positive and for HBsAg negative groups.

The JECFA estimated potency values for AFB1 which corresponded to 0.3 cancers year^−1^ 100,000^−1^ population/ng kg^−1^ bw day^−1^ (uncertainty range: 0.05–0.5) in HBs Ag positive individuals and 0.01 cancers year^−1^ 100,000^−1^ population/ng kg^−1^ bw day^−1^ (uncertainty range: 0.002–0.03) in HBsAg-negative individuals^11,39^ were adopted for this calculation. Also, the HBsAg + prevalence rate of 10.2% for Ghana^41^ was adopted and 89.8% (100–10.2%) was extrapolated for HBsAg-negative groups. Hence the average potency for cancer in Ghana was estimated as follows:

9 $$ \begin{aligned} {\text{Average}}\;{\text{ potency}} & = \left( {0.{3} \times 0.{1}0{2}} \right) + \left( {0.0{1} \times 0.{898}} \right) \\ & = 0.0{3958}\;{\text{cancers}}\;{\text{per}}\;{\text{ year}}\;{\text{per}}\;{1}00,000\;{\text{population}}\;{\text{ per}}\;{\text{ ng}}\;{\text{Aflatoxins }}\;{\text{kg}}^{{ - {1}}} {\text{bwday}}^{{ - {1}}} \\ \end{aligned} $$

Thus the population risk was estimated using the following formula:

10 $$ {\text{Population}}\;{\text{ risk}} = {\text{Exposure}}\; \, \left( {{\text{EDI}}} \right) \times {\text{Average}}\;{\text{ potency}} $$

### Statistical analysis

The aflatoxins concentrations were calculated using regression analysis from the curves derived from the standards of the aflatoxins with Excel for Microsoft Windows (version 10). Means and standard deviations of results were subjected to analyses of variance (one-way ANOVA) at the significant difference (p < 0.05) and separation of means were determined via post-hoc test using Duncan’s multiple range test DMRT with SPSS 22 (Chicago, USA). Means and standard deviations were computed and graphical representations were used appropriately.

## Results

Good linearity or coefficients of correlations (R^2^ > 0.990) within the tested range was obtained for most of the food samples tested. For the recovery analysis, one maize and rice samples were previously analyzed to assure the absence of studied mycotoxins, were used in the validation procedure. The Limits of Detection for AFB_1_ and AFB_2_ likewise AFG_1_ and AFG_2_ ranged between 0.13 and 0.15 while Limits of Quantification ranged between 0.26 and 0.30 respectively for both (Table 2).

The number of maize samples contaminated with AFB1, AFB_2_, AFG_1_, AFG_2_ and AF Total (Total Aflatoxins) are presented in Tables 3, 4, 5, 6 and 7. The order of toxicity was AFB_1_ > AFB_2_ > AFG_1_ > AFG_2_. As explained by Quinto et al.^42^ the terminal furan moiety of AFB_1_ is the critical point for determining the degree of biological activity of this group of fungal toxins. Out of a total of ninety (90) samples investigated, 72 (80%) tested positive for AFB_1_ and the contamination levels ranged from 0.78 ± 0.04 to 339.3 ± 8.6 µg kg^−1^ for MzNavrongo and MzEjura respectively. For AFB_2_, 59 (65.5%) samples tested positive and had levels ranging from 0.52 ± 0.02 to 103 ± 2.5 µg kg^−1^ for MzSefwi-Wiawso and MzEjura respectively. AFG_1_ was present in 35 (38.8%) samples of range 0.98 ± 0.03–14.5 ± 1.2 µg kg^−1^ for MzDzodze and MzBielepong respectively while, AFG2 was detected in only 14 (15.5%) samples, and their values ranged between 1.09 ± 0.03–5.51 ± 0.26 µg kg^−1^ for MzLawra and MzOffinso respectively. Lastly, a total ranged between 0.78 ± 0.04- 445.01 ± 8.9 µg kg^−1^ for MzNavrongo and MzEjura respectively. The total aflatoxin determinations were obtained from 72 (80.2%) samples.

**Table 3:** Concentration of aflatoxin types in different maize samples from different locations of Ghana.

Category	Food sample	Concentrations of Aflatoxins (µg kg^−1^)				
		AFB1	AFB2	AFG1	AFG2	Total
White	MzHalf- Assini	59.5 ± 0.99^a^	10.9 ± 0.4^a^	n.d	n.d	70.4 ± 1.8^a^
	MzDzodze	8.77 ± 0.47^ g^	2.40 ± 0.3^d^	0.98 ± 0.03^d^	n.d	12.15 ± 1.1^ h^
	MzKaneshie	16.02 ± 1.2^e^	2.53 ± 0.15^dc^	n.d	n.d	18.55 ± .0.9^ g^
	MzKsi cm	9.65 ± 0.25^ g^	2.98 ± 0.92^c^	2.71 ± 0.91^bc^	n.d	15.34 ± 0.9^gh^
	MzSuhum	16.27 ± 0.39^e^	3.11 ± 0.5^c^	1.89 ± 0.05^d^	1.97 ± 0.06^a^	23.24 ± 0.32f.
	MzKintamp	20.4 ± 0.8^d^	4.93 ± 0.27^bc^	3.51 ± 0.85b	1.66 ± 0.06^a^	30.5 ± 2.15^dc^
	MzMoree	8.03 ± 1.7^ g^	0.93 ± 0.04^e^	n.d	n.d	8.96 ± 0.22^i^
	MzKorle G	n.d	n.d	n.d	n.d	n.d
	MzNsawam	4.92 ± 0.6^ h^	0.69 ± 0.03^e^	n.d	n.d	5.61 ± 0.26^i^
	MzHo cm	n.d	n.d	n.d	n.d	n.d
	MzWa	n.d	n.d	n.d	n.d	n.d
	MzKotokraba	5.31 ± 0.26^ h^	0.86 ± 0.03^e^	n.d	n.d	6.17 ± 0.8^i^
	MzAowin	47.75 ± 2.25^b^	5.23 ± 0.25^b^	2.98 ± 0.9^b^	n.d	55.96 ± 1.2^b^
	MzSunyani	34.32 ± 2.1^c^	5.2 ± 0.22^b^	n.d	n.d	39.55 ± 2.3^dc^
	MzMadina	14.92 ± 0.5^ef^	1.5 ± 0.07^de^	2.31 ± 0.91^bc^	n.d	18.73 ± 0.65^ g^
	MzAsafo	42.07 ± 1.5^b^	3.17 ± 0.15^c^	n.d	n.d	45.24 ± 1.45^c^
	MzBerekum	5.18 ± 0.25^ h^	0.67 ± 0.02^e^	n.d	n.d	5.85 ± 0.26^i^
	MzTema	11.17 ± 1.22f.	10.24 ± 1.02^a^	5.6 ± 0.55^a^	n.d	27.01 ± 0.4^e^

Mean ± Standard Deviation that do not share a letter are significantly different (p > 0.05).

*n.d* not detected.

**Table 4:** Concentration of aflatoxin types in different maize samples from different locations of Ghana.

Category	Food sample	Concentrations of Aflatoxins (µg/kg)				
		AFB1	AFB2	AFG1	AFG2	Total
White	MzKoforidua	n.d	n.d	n.d	n.d	n.d
	MzWalewale	12.87 ± 1.4^c^	0.61 ± 0.05^e^	n.d	n.d	13.48 ± 0.35^d^
	MzNsawkaw	5.29 ± 0.24^de^	n.d	2.51 ± 0.14^b^	n.d	7.8 ± 0.45^e^
	MzAsamankese	7.44 ± 1.2^d^	4.86 ± 0.44^b^	4.22 ± 0.2^a^	2.06 ± 0.17^a^	18.58 ± 1.1^c^
	MzHohoe	14.4 ± 1.26^c^	0.56 ± 0.03^e^	n.d	n.d	14.96 ± 1.3^d^
	MzAbutia	7.07 ± 0.32^d^	0.52 ± 0.02^e^	n.d	n.d	7.53 ± 0.50^e^
	MzSogakope	12.92 ± 1.2^c^	5.0 ± 0.42^b^	4.2 ± 0.26^a^	n.d	22.12 ± 0.45^c^
	MzKintampo	n.d	n.d	n.d	n.d	n.d
	MzTamale	44.20 ± 0.51^a^	7.7 ± 0.50^a^	1.6 ± 0.05^bc^	n.d	53.5 ± 0.95^a^
	MzKayera	n.d	n.d	n.d	n.d	n.d
	MzNanton1	10.63 ± 1.2^c^	n.d	n.d	n.d	10.63 ± 1.20^d^
	MzNanton 2	11.42 ± 1.22^c^	1.19 ± 0.1^d^	n.d	n.d	12.61 ± 1.20^d^
	MzSavelugu	22.33 ± 0.35^b^	4.75 ± 0.48^bc^	n.d	n.d	27.08 ± 1.4^b^
	MzAssin fosu	20.73 ± 0.3^b^	4.75 ± 0.48^bc^	n.d	n.d	25.08 ± 1.4^b^
	MzAdeamra	n.d	n.d	n.d	n.d	n.d
	MzAfrancho	n.d	n.d	n.d	n.d	n.d
	MzEjisu.mkt	8.55 ± 0.60^d^	n.d	n.d	n.d	8.55 ± 0.60^e^
	MzAfrmso	4.96 ± 0.43^e^	n.d	2.09 ± 0.13^b^	n.d	7.05 ± 0.50^e^

Mean ± Standard Deviation that do not share a letter are significantly different (p > 0.05).

*n.d* not detected.

**Table 5:** Concentration of aflatoxin types in different maize samples from different locations of Ghana.

Category	Food sample	Concentrations of Aflatoxins (µg/kg)				
		AFB1	AFB2	AFG1	AFG2	Total
White	MzDonkork	60.51 ± 1.65^c^	17.66 ± 1.5^c^	10.21 ± 1.1^b^	2.7 ± 0.6^c^	91.08 ± 2.1^b^
	MzKwaekese	7.86 ± 0.45^ h^	0.54 ± 0.04^ g^	n.d	n.d	8.4 ± 0.85^ g^
	MzPedu	n.d	n.d	n.d	n.d	n.d
	MzAfrm.pl	14.28 ± 1.20^ g^	1.58 ± 0.06^ g^	13.19 ± 0.9^a^	n.d	29.05 ± 3.50f.
	MzKayeru	n.d	n.d	n.d	n.d	n.d
	MzKamina	2.5 ± 0.12^i^	n.d	4.53 ± 0.22^d^	n.d	7.03 ± 0.4^ g^
	MzAgbogbloshi	45.13 ± 0.61^d^	16.32 ± 1.3^c^	11.5 ± 1.22^b^	2.8 ± 0.12^c^	75.75 ± 1.84^c^
	MzNima	39.5 ± 3.01^de^	13.77 ± 0.9^d^	4.99 ± 0.26^d^	5.5 ± 0.27^a^	63.76 ± 1.71^d^
	MzMakola	94.42 ± 2.24^b^	n.d	n.d	n.d	94.42 ± 2.24^b^
	MzZuo	1.88 ± 0.06^i^	n.d	n.d	n.d	1.88 ± 0.06^ h^
	MzZebilla	2.26 ± 0.6i	n.d	n.d	n.d	2.26 ± 0.6^ h^
	MzMankesim	23.11 ± 0.31f.	8.3 ± 0.5f.	4.67 ± 0.47^d^	n.d	36.08 ± 2.5^e^
	MzSandema	43.7 ± 2.25^d^	21.6 ± 0.33^b^	13.75 ± 1.2^a^	n.d	79.05 ± 1.82^c^
	MzNkwatia	232.47 ± 8.33^a^	15.77 ± 1.4^c^	n.d	n.d	248.24 ± 8.45^a^
	MzDromkma	n.d	n.d	n.d	n.d	n.d
	MzFunsi	36.4 ± 2.5^de^	20.5 ± 0.35^b^	6.8 ± 0.33^ cd^	1.11 ± 0.05^d^	64.8 ± 1.67^d^
	MzAbliri	25.2 ± 0.35f.	11.7 ± 1.2^de^	n.d	n.d	36.9 ± 2.14^e^
	MzBielepong	51.73 ± 0.3^d^	24.1 ± 0.4^a^	14.5 ± 1.2^a^	3.5 ± 0.6^b^	93.83 ± 5.2^b^

Mean ± Standard Deviation that do not share a letter are significantly different (p > 0.05).

*n.d* not detected.

**Table 6:** Concentration of aflatoxin types in different maize samples from different locations of Ghana.

Category	Food Sample	Concentrations of Aflatoxins (µg/kg)				
		AFB1	AFB2	AFG1	AFG2	Total
White	MzBakano	n.d	n.d	n.d	n.d	n.d
	MzUCC	2.2 ± 0.15^i^	n.d	n.d	n.d	2.2 ± 0.15^j^
	MzAdu1	n.d	n.d	n.d	n.d	n.d
	MzAdu2	n.d	n.d	n.d	n.d	n.d
	MzEjura	339.3 ± 8.6^a^	103 ± 2.5^a^	2.71 ± 0.12^ cd^	n.d	445.01 ± 8.9^a^
	MzBonwire	102.1 ± 2.5^c^	0.88 ± 0.03^de^	n.d	n.d	102.98 ± 2.5^d^
	MzSefw. W	11.5 ± 0.3^ g^	0.62 ± 0.03^de^	n.d	n.d	12.12 ± 1.4^ g^
	MzBibiani	5.21 ± 0.32^ h^	1.5 ± 0.06^d^	n.d	n.d	6.71 ± 0.08^i^
	MzSefw.Bk	185.17 ± 2.15^b^	9.37 ± 0.25^b^	n.d	n.d	194.54 ± 2.5^b^
	MzAtwim	18.21 ± 1.31f.	2.66 ± 0.13^c^	n.d	n.d	20.87 ± 0.35^ g^
	MzT’di	3.93 ± 0.22^ h^	0.79 ± 0.03^de^	n.d	n.d	4.72 ± 0.28^i^
	MzNsaw	n.d	n.d	n.d	n.d	n.d
	MzTumu	75.4 ± 1.85^d^	40.3 ± 0.55^a^	7.62 ± 0.33^b^	2.57 ± 0.3^b^	125.89 ± 2.5^c^
	MzAzuguyeri	24.7 ± 0.35^e^	6.77 ± 0.30^bc^	2.61 ± 0.92^ cd^	n.d	34.08 ± 3.01f.
	MzBolga	5.18 ± 0.25^ h^	2.91 ± 0.9^c^	1.02 ± 0.02^d^	n.d	9.11 ± 0.22^ h^
	MzKayeru	25.96 ± 0.31^e^	7.89 ± 0.32^b^	1.22 ± 0.06^d^	n.d	35.07 ± 2.82f.
	MzChogu	10.91 ± 1.22^ g^	4.23 ± 0.23^c^	2.5 ± 0.92^ cd^	1.88 ± 0.06^bc^	19.52 ± 0.31^ g^
	MzOffinso	27.78 ± 0.52^e^	9.47 ± 0.46^b^	5.41 ± 0.25^ab^	5.51 ± 0.26^a^	48.17 ± 0.55^e^

Mean ± Standard Deviation that do not share a letter are significantly different (p > 0.05).

*n.d* not detected.

**Table 7:** Concentration of aflatoxin types in different maize samples from different locations of Ghana.

Category	Food Sample	Concentrations of Aflatoxins (µg/kg)				
		AFB1	AFB2	AFG1	AFG2	Total
Colored	MzLawra	2.69 ± 0.12^e^	1.55 ± 0.05^ab^	1.01 ± 0.02^b^	1.09 ± 0.03^a^	6.29 ± 0.81^ cd^
	MzDambai	5.35 ± 0.7^d^	1.67 ± 0.05^ab^	1.05 ± 0.11^b^	n.d	8.07 ± 0.85^c^
	MzGbawe	2.37 ± 0.12^e^	n.d	n.d	n.d	2.37 ± 0.12^e^
	MzKintampo	16.95 ± 0.84^a^	1.04 ± 0.06^ab^	3.67 ± 0.22^a^	n.d	21.66 ± 0.32^a^
	MzGurugu	1.77 ± 0.06^e^	n.d	n.d	n.d	1.77 ± 0.06^e^
	MzKeta	1.61 ± 0.06^e^	n.d	n.d	n.d	1.61 ± 0.06^e^
	MzTech jxn	0.88 ± 0.02^ef^	0.67 ± 0.02^ab^	n.d	n.d	1.55 ± 0.06^e^
	MzGwolu	1.78 ± 0.06^e^	0.80 ± 0.03^ab^	n.d	n.d	2.66 ± 0.15^e^
	MzDormaa	3.01 ± 0.5^e^	2.55 ± 0.14^a^	n.d	n.d	5.56 ± 0.26^ cd^
	MzTechiman	17.43 ± 0.81^a^	3.2 ± 0.22^a^	n.d	n.d	20.63 ± 0.33^a^
	MzBame	5.2 ± 0.5^c^	0.77 ± 0.04^ab^	1.02 ± 0.05^b^	n.d	6.99 ± 0.82^c^
	MzKwadaso	8.41 ± 0.85^b^	2.85 ± 0.14^a^	1.01 ± 0.05^b^	n.d	11.26 ± 1.22^b^
	MzLapaz	4.5 ± 0.27^d^	1.6 ± 0.07^ab^	1.07 ± 0.06^b^	n.d	7.17 ± 0.45^c^
	MzJuaboso	1.89 ± 0.06^e^	0.82 ± 0.04^ab^	n.d	n.d	2.71 ± 0.15^e^
	MzNavrongo-ue	0.78 ± 0.04^ef^	n.d	n.d	n.d	0.78 ± 0.04f.
	MzMkt circle	n.d	n.d	1.1 ± 0.02^b^	n.d	1.1 ± 0.02^e^
	MzAnomabo	n.d	n.d	n.d	n.d	n.d
	MzNadowli	n.d	n.d	n.d	n.d	n.d

Mean ± Standard Deviation that do not share a letter are significantly different (p > 0.05).

*n.d* not detected.

The greatest aflatoxin yields of 445.01 ± 8.9 and 339.3 ± 8.6 µg kg^−1^ for AF_Total_ and AFB_1_ respectively, were significantly (p < 0.05) higher than all other samples studied in the various categories.

Toxin quantity limits prescribed by the Ghana Standards Authority which are slightly higher and more flexible than the European Union (EFSA) are 5, 10 µg kg^−1^ and 2, 4 µg kg^−1^ respectively for AFB_1_ and Total aflatoxins.

Results from the study were compared to the European Food Safety Authority (EFSA) and Ghana Standards Authority (GSA) regulatory concentration limits for total aflatoxins (AF Total) and Aflatoxins B_1_ (AFB_1_). From Table 8, a majority of 41/72 (56.9%) of the 72 samples analyzed for total aflatoxins in group one (white maize samples) exceeded the limits of GSA. These maize samples had AF_Total_ ranging from 12.12 ± 1.4 to 445.01 ± 8.9 µg kg^−1^. Only 3/18 (16.67%) out of colored maize samples (Group two) was found to exceed the GSA limit which also ranged 11.26 ± 1.22–21.66 ± 0.32 µg kg^−1^.

**Table 8:** Proportions of samples that exceeded AF_Total_ and AFB_1_ and limits of Ghana Standard Authority (GSA) and the European Food Safety Authority (EFSA).

Samples	Total samples	Exceeding GSA regulation		Exceeding EFSA regulation	
		Yes (%)	Range	Yes (%)	Range
**AFTotal**					
Group 1	72	41 (56.9%)	12.12 ± 1.4–445.01 ± 8.9	54 (75%)	4.72 ± 0.28–445.01 ± 8.9
Group 2	18	3 (16.67%)	11.26 ± 1.22–21.66 ± 0.32	8 (44.4%)	5.56 ± 0.26–21.66 ± 0.32
Total	90	47 (52.2%)	11.26 ± 1.22–445.01 ± 8.9	62 (68.89%)	4.72 ± 0.28–445.01 ± 8.9
**AFB1**					
Group 1	72	50 (69.44%)	5.18 ± 0.25–339.3 ± 8.6	56 (77.78%)	2.2 ± 0.15–339.3 ± 8.6
Group 2	18	5 (27.78%)	5.2 ± 0.5–17.43 ± 0.81	9 (50.0%)	2.37 ± 0.12–16.95 ± 0.84
Total	90	55 (60.85%)	5.18 ± 0.25–339.3 ± 8.6	65 (72.2%)	2.2 ± 0.15–339.3 ± 8.6

European Union Food Safety (EFSA) limit for AF_Total_ = 4 µg kg^−1^.

European Union Food Safety (EFSA) limit for AFB1 = 2 µg kg^−1^.

Ghana Standards Authority (GSA) limit = 10 µg kg^−1^.

Ghana Standards Authority (GSA) limit = 5 µg kg^−1^.

Overall, 47/90 (52.2%) of the maize recorded values above the set limit and ranged 11.26 ± 1.22–445.01 ± 8.9 µg kg^−1^.

About 54/72 (75%) of white maize and 8/18 (44.4%) colored maize corresponding to ranges of 4.72 ± 0.28–445.01 ± 8.9 and 5.56 ± 0.26–21.66 ± 0.32 µg kg^−1^ respectively exceeded the tolerable limit of the EFSA. Overall for EFSA, 62/90 (68.89%) of white maize samples of range 4.72 ± 0.28–445.01 ± 8.9 µg kg^−1^ exceeding limits were recorded. Whereas 70.4 and 11.5% of samples respectively from the two groups exceeded EFSA for AFB_1_ (Table 8).

For AFB_1_, GSA limits exceeded were 50/72 (69.44%) and ranged 5.18 ± 0.25–339.3 ± 8.6 µg kg^−1^ while for the colored maize samples, 5/18 (27.78%) of range 5.2 ± 0.5–17.43 ± 0.81 µg kg^−1^ were recorded. For the overall, 55/90 (60.85%) of range, 5.18 ± 0.25–339.3 ± 8.6 µg kg^−1^ were recorded.

Values of 56/72 (77.78%) with range 2.2 ± 0.15–339.3 ± 8.6 µg kg^−1^ and 9/18 (50.0%) with range 2.37 ± 0.12–16.95 ± 0.84 µg kg^−1^ were recorded for white and colored maize samples respectively. Finally, as the overall, 65/90 (72.2%) maize samples of range 2.2 ± 0.15–339.3 ± 8.6 µg kg^−1^ exceeded limits for EFSA.

### Risk assessment

The Estimated Daily Intakes (EDI) of the total aflatoxins in the maize samples were 109.7, 58.8, 33.08 and 25.2 μg Kg bw^−1^ day^−1^ for infants, children, adolescents, and adults respectively. For the Hazard Quotient (HQ), values of 5.5 × 10^5^, 2.94 × 10^5^, 1.65 × 10^5^, and 1.26 × 10^5^ were recorded respectively. A range of 2.5–10.97 was recorded for H.I. Margin of Exposure (MOE) values recorded were 3.64, 6.80, 12.09 and 6.75 respectively. The average potency of the aflatoxins was 0.0396 ng Aflatoxins kg^−1^ bw day^−1^ and produced a population risks of 4.344, 2.3, 1.31 and 1.0 respectively (Table 9).

**Table 9:** Evaluation of risk for Total Aflatoxins via consumption of maize.

	Av.body wgt.(kg)	References (weight)	Estimated daily intake (EDI) (μg Kg bw^−1^ day^−1^)	Hazard quotient (H.Q)	hazard index (h.i)	MOE	Av. potency (ng Aflatoxins kg^−1^ bw day^−1^)	Population risk
Infants (6–52mths)	7	Glover-Amengor et al.^43^ Abeshu et al.^44^	109.7	5.5 × 10^5^	10.97	3.64	0.0396	4.344
Children (5–11 years)	26 (24–28)	Biritwum et al.^45^ WHO^46^	58.8	2.94 × 10^5^	5.88	6.80	0.0396	2.3
Adolescents (12–18 years)	46.25 (38.5–54)	Afrifa-Anane et al.^47^	33.08	1.65 × 10^5^	3.30	12.09	0.0396	1.31
Adults (18–60 years)	60.7	Walpole et al.^48^	25.2	1.26 × 10^5^	15.87	6.75	0.0396	1.0

Margin of Exposure-MOE.

*Mean aflatoxins-36.13 µg kg^−1^.

*Daily intake of maize for infants was halved (0.5 × 42.5 kg).

*Daily intake of 42.5 kg was used for children, adolescents, and adults.

TD_50_ = 0.2 ng Kg bw^−1^^49^.

1 ng = 0.001 μg.

For AFB_1_, the EDIs for infants, children, adolescents, and adults were 84.5, 45.5, 25.6 and 19.5 μg Kg bw^−1^ day^−1^ respectively. HQ values recorded were 5.5 × 10^5^, 2.94 × 10^5^, 1.65 × 10^5^ and 1.26 × 10^5^ respectively. Margin of Exposure (MOE) values recorded were 4.73, 8.79, 15.63 and 20.51 respectively. The average potency was the same as total aflatoxins while the population risks were respectively 3.35, 1.80, 1.01 and 0.77 (Table 10).

**Table 10:** Evaluation of risk for Aflatoxins B1 (AFB1) via consumption of maize.

	Av.Body weight (kg)	References (weight)	Estimated daily intake (EDI) (μg Kg bw^−1^ day^−1^)	Hazard quotient (H.Q)	Hazard Index (H.I)	MOE	Av. potency (Aflatoxins ng kg^−1^ Bw day^−1^)	Population risk
Infants (6–52mths)	7.08 (2.5–11.65)	Glover-Amengor et al.^43^ Abeshu et al.^44^	84.5	4.2 × 10^5^	8.5	4.733	0.0396	3.35
Children (5–11 years)	26 (24–28)	Biritwum et al.^45^ WHO^46^	45.5	2.3 × 10^–3^	4.6 × 10^–3^	8.79	0.0396	1.80
Adolescents (12–18 years)	46.25 (38.5–54)	Afrifa-Anane et al.^47^	25.6	1.3 × 10^5^	2.6	15.63	0.0396	1.01
Adults (18–60 years)	60.7	Walpole et al.^48^	19.5	9.7 × 10^4^	1.95	20.51	0.0396	0.77

The margin of Exposure- MOE.

TD_50_ = 0.2 ng kg bw^−1^^49^.

1 ng = 0.001 μg.

*Mean of AFB1 27.85 µg kg^−1^.

*Daily intake of maize for infants was halved (0.5 × 42.5).

*Daily intake of 42.5 kg was used for children, adolescents, and adults.

## Discussion

Aflatoxin contamination of market maize is a significant public health concern. Our findings demonstrated widespread aflatoxin contamination of maize within the regional market distribution system. The different maize samples obtained from the different markets across the country had varying quantities of AFB1 and AFtotal. The greatest quantity of aflatoxins recorded from this study (445.01 ± 8.9 µg kg^−1^) was, by and large, lower than values of 692 and 945 ng g^−1^ from maize samples obtained from Fumesua and Ejura in Ghana respectively by Dadzie et al.^24^.

Kpodo^50^ in earlier surveys reported aflatoxin levels in the range of 20–355 ng g^−1^ maize samples from silos and warehouses in Ejura while fermented corn dough collected from major processing sites also contained aflatoxin levels of 0.7–313 ng g^−1^. James et al.^51^ also found high average aflatoxin levels in maize samples collected from North Kwahu (153 ng g^−1^), Ejura Sekyere-Dumasi (121 ng g^−1^) and Nkoranza (134 ng g^−1^).

Agbetiameh et al.^26^ reported values of range 1–341 ppb in maize from different ecological zones in Ghana. Likewise, Blankson et al.^28^ also reported a range of 1.77 ± 0.01–24.58 ± 0.05 μg kg^−1^ in maize-based samples in locally prepared cereals for consumption in Ghana.

From Kenya, Nduti et al.^52^ reported values of range 7.92 ± 1.57–22.54 ± 4.94 ppb from maize flour samples obtained from three regions.

Lewis et al.^8^ reported greater values of aflatoxin quantities of > 1000 ppb from maize samples as they investigated aflatoxin contamination of commercial maize products during an outbreak of acute aflatoxicosis in Eastern and Central Kenya.

AFB1 was present in seven of eight samples ranging from 30 to 6127 μg kg^−1^ and two of eight samples were found positive for AFB2 ranging from 53 to 1738 μg kg^−1^ as reported by Sewram et al.^53^ from Brazil as they investigated corn-based infant food sold on different markets. In their study in Ghana, aflatoxins were reported to markedly contaminate local food (weanimix) from cereal-legume blend for children and reported aflatoxin B1 levels in local weanimix of range 7.9–500 ppb (ug kg^−1^)^54^.

The reasonably high levels of aflatoxins detected in this study, put forward that aflatoxins can continue to persist in food even after the inactivation of the fungi in spite of all the rigorous processing methods because of their ability to resist chemical and thermal changes^55^. A danger looms hereafter, since there is a high likelihood of detecting aflatoxins in processed cereal foods more so if the ingredients used for the food are initially contaminated before processing and subsequent consumption. Thus, the incidence of aflatoxins in processed cereal-based food might indicate contamination of the raw cereals at a point in the value chain (either on the farm or during storage). It is also noteworthy that the not detected (n.d) status recorded in some samples may not necessarily mean total absence of aflatoxins but were simply below detectable limits of the equipment.

Aflatoxins contamination occurs via an initial phase during crop development and a second phase during crop maturation. The contamination is greater in warm, humid, and even hot deserts and drought conditions^56^ since mycotoxins are optimally produced in adverse periods.

Atoxigenic biocontrol of aflatoxins promises a safe, economical, ecosystem friendly, cost-effective method of aflatoxin mitigation throughout the value chain^57–61^. Implementing aflatoxin biocontrol management strategies to reduce aflatoxin contamination in the field and throughout storage would result in improved health, enhanced trade, increased income, and the welfare of farmers and consumers in Ghana.

Climatic variations of the different agro-ecological zones might be an explanation to the seasonal occurrences of types and levels of toxins in the food material^62^. Planning interventions against dietary exposure to aflatoxins form an important basis for these current findings since the rainfall pattern in major parts of the country is categorized bimodally by long and short rainy seasons intermingled with brief dry spells^62^. A general mycotoxicosis risk is signaled through a seasonal assessment of aflatoxin residues in food specifically given climatic changes triggering hot and dry influences linked to increased contamination^56,63,64^. It was also observed in this study that colored maize recorded lower quantities of aflatoxins as compared with levels of the white maize samples. Presumably, the biochemical substances present in the maize accounting for the colorations such as beta carotene, zeaxanthins, lutein etc. may have inhibited the propagation of *Aspergillus* species.

In Africa, of which Ghana is no exception, smallholder farmers produce most of the maize of which most of them use poor harvest techniques such as insufficient drying and storage of their crops. Additionally, the cultivation of local maize varieties which are susceptible to both insect damage and diseases, less drought-tolerant, expose the maize to infection by encouraging the growth of aflatoxin-producing fungi during crop development, maturation and harvest in the field^65^.

Despite the availability of improved varieties, local varieties are still planted by a significant fraction of maize farmers. Even though there is knowledge of the occurrence of aflatoxin accumulation in maize collected in markets and farmer stores across Ghana^26^, little is known of the aflatoxin levels when the maize is still in the field (prior-to-harvest maize) and the composition of community structures of *Aspergillus* section *flav*or associated with the maize in Ghana.

## Conclusion

It can be surmised from the results of this study that from a total of ninety (90) maize samples investigated, 65 (72.2%) tested positive for AFB_1_ and ranged from 2.2 ± 0.15–339.3 ± 8.6 µg kg^−1^. A similar proportion of 62 (68.89%) was also recorded for total aflatoxins (AF_Total_) and ranged between 4.72 ± 0.28–445.01 ± 8.9 µg kg^−1^ and these were above the limits set by the Ghana Standards Authority (GSA) (5 and 10–15 µg kg^−1^) and the European Food Safety Authority (EFSA) (2 and 4 µg kg^−1^). Human health risk assessment from aflatoxins exposure through maize consumption from the markets by infants, children, adolescents, and adults showed a significant non-carcinogenic adverse health risk to humans since all calculated values for Hazard Quotient (HQ) were > 1 while there was no observed adverse health effect (HI < 1). Conversely, MOE values obtained in this study were all greater than 0.04 ngkg^−1^ bw day^−1^ which implied a potential health risk as suggested by the World Health Organization (2008).

Government institutions, private and non-governmental organizations, as well as national media networks need to play a key role in raising consciousness of the public health impacts of aflatoxin. Bearing in mind the necessity of attaining food security and food safety for vulnerable people in these areas, there is also a need for data and risk management capacity tools for locally driven policy reform.

### Limitations of the study

The human health risk assessments were arrived at using deterministic methods (based on assumptions) as opposed to using probabilistic approaches.

The primary data obtained from the laboratory, are usually presented as means + standard deviation from three determinations which statistically, do not represent the true average of the concentrations.
